# Autopsy results from a COVID-19 patient treated in a tropical area, and a review of the epidemiological history

**DOI:** 10.1080/20961790.2021.1978173

**Published:** 2022-03-03

**Authors:** Jie Cai, Bo Wang, Tao Song, Peng Zhang, Ren Long, Xiaoran Liu, Jianqiang Deng, Jianhua Chen

**Affiliations:** a Department of Forensic Medicine, Hainan Tropical Forensic Engineering Research Center, Hainan Medical University, Haikou, China; b Key Laboratory of Emergency and Trauma of Ministry of Education, Emergency and Trauma College, Hainan Medical University, Haikou, China

**Keywords:** Forensic sciences, forensic pathology, COVID-19, SARS-CoV-2, autopsy, tropical area, epidemiological history

## Abstract

Since the start of the COVID-19 pandemic, there has been an urgent need to produce accurate and sensitive tests. However, there have been instances where a positive nucleic acid test turns negative after treatment, and then positive again. This case report describes such an instance from the tropical region of Hainan, China. The patient was a 61-year-old female who went to Hainan on vacation from Wuhan during the COVID-19 pandemic in 2020. Symptoms appeared 9 d after arriving in Hainan, and it was confirmed that the nucleic acid test was positive after 4 repeats. Her condition declined rapidly, her heart stopped beating, and she was admitted in a coma to the ICU. After treatment, the SARS-CoV-2 virus nucleic acid test of several nasopharyngeal swabs were negative, and tests on whole blood, anal swabs, and urine were also negative. Later, however, nucleic acid tests on a lower respiratory tract sputum swab and lower respiratory tract lavage fluid were positive. An autopsy examination was carried out 12 h after her death, and multi-organ secretions were extracted for nucleic acid testing. The SARS-CoV-2 virus nucleic acid was only detected in the swabs from the end of the bronchus, which was confirmed by the visualization of the coronavirus by electron microscopy. Autopsy confirmed that the damage was mainly concentrated in the lungs and immune organs and tissues throughout the body. Epidemiology indicated that none of the people she came into contact with after arriving in Hainan, including close contacts, were infected. This is in sharp contrast to the highly contagious virus in Wuhan in the temperate zone during the same period. This case report indicates: (1) The high temperatures in tropical areas may have an impact on the spread and harm of COVID-19, and (2) The reason why nucleic acid testing for COVID-19 was initially negative and then positive after treatment may be related to the survival of the SARS-CoV-2 virus in deep lung tissues.

## Introduction

The global pandemic of COVID-19 in 2020 has brought unprecedented threats and challenges to human health and life. Scientists from numerous countries have investigated the new coronavirus (SARS-CoV-2), and how it affects human health. One particularly important line of investigation has been autopsy investigations of patients with new coronavirus. Such investigations can provide first-hand, direct, and accurate data on the damage to the human body by the disease [1–4]. The medical community recognizes that the occurrence and development of diseases in tropical regions have different characteristics from other regions, but there are still very few studies on COVID-19 in tropical regions, and more cases are needed [5–7]. Hainan Province, located on Hainan Island, is the only province in China that is in the tropical region. This COVID-19 patient died after the onset of the disease in Hainan Province, and was the only patient from whom pathological anatomy was obtained after dying in the tropical region of China. She was also the only patient with COVID-19 for whom pathological anatomy was obtained outside of Wuhan, China. The anatomical findings and epidemiological history of this patient can increase our understanding of COVID-19 in tropical areas and provide direct and reliable data for related research.

### Case history

The patient is a 61-year-old female who lived in Wuhan and arrived in Hainan on Jan 19, 2020. She developed fever, chills, cough and fatigue on Jan 27. On Jan 29, her symptoms worsened and she was admitted to the hospital for treatment. Chest X-rays showed progressive infiltration and diffuse ground glass shadow in both lungs, and the left lung was especially obvious. Subsequently, she was subjected to three throat swab nucleic acid tests every other day, all of which were negative. On Feb 6, she suddenly experienced breathing difficulties, accompanied by wheezing, shortness of breath, cardiac arrest, and then coma. The hospital again took a nasopharyngeal swab sample and sent it for examination. The first test confirmed that the patient was infected with the SARS-CoV-2 virus and she was immediately sent to a designated hospital for treatment. Due to the seriousness of her condition, she was given tracheal intubation, ventilator-assisted breathing, antivirals interferon α-1 b and Lopinavir/Ritonavir tablets, and Moxifloxacin to prevent secondary infections. On Feb 9, the patient’s heart rate dropped from 102 beats/min to 51 beats/min, blood pressure gradually dropped to 50/30 mmHg, and respiratory rate is 13 breaths per minutes. After the patient had been stabilized, the blood pressure gradually rose to 100/62 mmHg, and the heart rate recovered to 97 beats/min. However, the patient was in a comatose state, with pupils in both eyes being equal circles with a diameter of 5 mm, and the light reflection disappeared. Methylprednisolone sodium succinate, Xuebijing, and continuous renal replacement therapy (CRRT), etc. were used to reduce lung inflammation, but the persistent coma and hypoxemia did not improve significantly. She received extracorporeal membrane oxygenation (ECMO) treatment on Feb 16, but her blood oxygen saturation remained around 85%. On Feb 20, 22, and 26, SARS-CoV-2 virus nucleic acid tests from nasopharyngeal swabs were negative. On Feb 26 the additional tests of whole blood, anal swabs, and urine were also negative. However, the nucleic acid tests taken from lower respiratory tract sputum swab samples and lower respiratory lavage fluid on Mar 1 and 3, respectively, showed positive results. On Mar 5 (the 39th day from onset), the patient suffered a cardiac arrest. After receiving active measures such as invasive ventilation, chest compression, and epinephrine injection, the rescue was not successful. She died at 9:20 on Mar 5, Beijing time. The family agreed the study with written informed consent.

### Forensic pathological examination

#### Forensic autopsy

**Figure 1.**
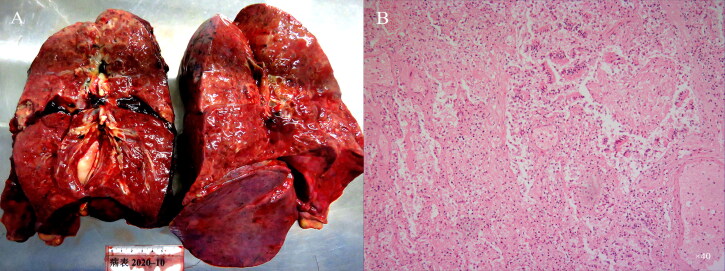
Cross-section of both lungs and view under the microscope. (A) The cut surface of the lungs showed consolidation and the formation of abscess cavity in the upper lobe of the left lung. (B) Histopathology found that the structure of lung tissue was destroyed, the remaining alveolar tissue was infiltrated by a large number of inflammatory cells, the small bronchial epithelium fell off, and the lumen was filled with mucus-like material (HE, ×40).

The autopsy was performed 12 h after the clinical diagnosis of her death. The main positive findings were: mild cyanosis of the lips. The larynx was moderately oedematous, and a small amount of slightly thick purulent discharge was seen in the throat. Dark red effusion (450 mL) was seen in the left chest cavity, and a large blood clot was seen on the back of the chest cavity. Light red clear liquid (150 mL) was seen in the right chest cavity. Scattered bleeding points were seen on the front and back sides of the heart surface. Light red effusion (350 mL) was seen in the abdominal cavity. A cloudy, slightly viscous bloody secretion and mucosal oedema were seen in the trachea and bronchi. The left lung weighed 650 g and the right lung 700 g. The lungs were full, the texture extensively hardened, and the chest wall extensively adhered to, and blood clots adhered to the lower lobe of the left lung. The cut surface of both lungs was highly oedematous, consolidation and turbidity, and more purulent secretions were seen locally. Multiple abscesses were formed in both lungs, the largest being 2.5 cm × 2.0 cm × 1.0 cm (Figure 1A). The remainder of the general examination showed no abnormalities.

#### Tissue microscopic examination

The internal organs and tissues of the whole body were examined and it was found that the main lesions were concentrated in the lungs and lymphatic organs.

#### Lungs

The lung showed a thickened capsule, congestion and oedema in the interstitium of both lungs, diffuse destruction of alveolar structure, unclear structure, widening of the remaining alveolar compartment, type II alveolar cell proliferation, and lack of hyaline membrane formation in the alveolar cavity. In the remaining alveolar cavity, serous, fibrinous and massive inflammatory cells exuded, mainly neutrophils and mononuclear macrophages, scattered in monocytes and lymphocytes. Multiple large and small abscesses were seen, and the surrounding fibrous tissue proliferated and was wrapped with a large number of neutrophil infiltrations, local vascular hyperplasia, congestion, and banded haemorrhage were seen around, and the remaining lung tissue structure was still seen in the middle of some abscesses. Most of the small bronchial walls had collapsed, glands slightly proliferated, scattered in mononuclear, lymphocyte infiltration, more neutrophil infiltration, a large number of mucus-like substances and shed epithelial cells in the lumen. Hilar lymph nodes were atrophied, the number of intranodal Hilar lymph nodes were atrophied, the number was reduced, and the density of lymphocyte distribution was reduced (Figure 1B).

#### Lymphatic organs and tissues

The lymph nodes in the spleen, tonsils, hilar lymph nodes and gastrointestinal tissues basically disappeared, lymphocytes were sparse and the number was reduced.

### Laboratory examination

#### SARS-CoV-2 virus nucleic acid detection in fixed organs and tissues

SARS-CoV-2 virus nucleic acid detection was performed on sections which had been treated with glutaraldehyde electron microscope preservation solution and formalin-fixed for 14 d. No viral nucleic acid was detected. The negative and positive controls were normal.

#### Electron microscopy of lung tissue

**Figure 2.**
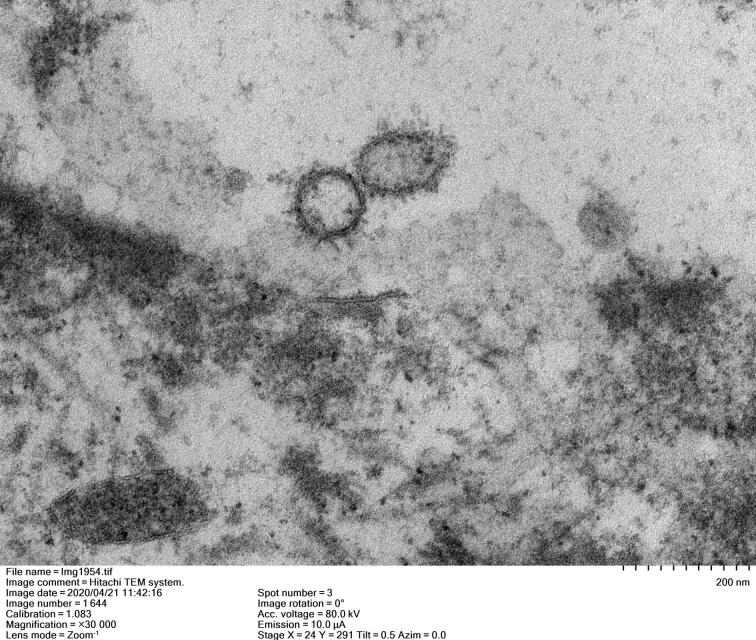
Coronavirus was detected in lung tissue by electron microscope.

During the autopsy, swabs were taken of cerebrospinal fluid, pleural effusion, blood, abdominal effusion, bile, and secretion from the trachea, stomach, intestine, and the end of the small bronchus of the lower lobe of the right lung. These were used for SARS-CoV-2 viral nucleic acid detection. The results showed that the only positive SARS-CoV-2 nucleic acid test was from the small bronchial end secretions of the lower lobe of the right lung. The lung tissue was observed under an electron microscope and coronavirus was found (Figure 2).

#### Special staining of lung tissue

Masson staining and AB-PAS staining indicated that the alveolar cavity was filled with a large amount of exuded protein mucus; containing neutral and acidic proteins. PAS staining and hexamine silver showed focal fungal hyphae and spore structures.

#### Immunohistochemical detection of lung tissue

CD3, CD4, CD8, CD20, CD138, CD68, and CD56 tests found that hilar lymph nodes and lymph follicles were atrophied, the number decreased, and the number of various immune cells significantly decreased.

## Discussion

When this patient departed from Wuhan, it was at the beginning of the COVID-19 outbreak. Subsequently, the well-known COVID-19 pandemic occurred which was found to be extremely contagious in Wuhan. According to current epidemiological investigations, the average incubation period of COVID-19 is 5 d, with a range of 2–14 d [8]. In this case, the patient arrived in Hainan the day after leaving Wuhan. Symptoms began to appear on the 9th day after arriving in Hainan. She had no history of contact with COVID-19 patients in Hainan. Since the disease had spread to a large area when she left Wuhan, and her onset time was also within the incubation period from when she left Wuhan, the source of the virus was determined to have been in Wuhan. The most prominent feature of this case is that all the people in close contact with the patient after arriving in Hainan, including her fellow travelers, co-residents, and other close contacts, were more than five people, and since her condition was unknown she attended the outpatient clinic of the hospital. At that time, the medical staff had only routine protection and the patients in the same clinic had no infection, which is in sharp contrast with the spread of the disease in Wuhan.

At present, it is generally believed that the climatic differences between tropical regions and other regions have an important impact on the spread of viruses (such as influenza, coronavirus, etc.), the survival of the virus in the host, and the immunity of the host population. Meteorological conditions include temperature, humidity, wind speed and direction, atmospheric pressure, solar radiation, etc. Temperature and humidity are the factors thought to have the greatest impact on the spread of the virus [9–11]. Cold weather conditions are conducive to better survival of the virus outside the human body, leading to increased transmission. Conversely, cold air cools the epithelial cells of the nasal cavity, thereby reducing the mechanical defence capabilities of the respiratory and immune systems. After the outbreak of COVID-19 in 2020, there were also some epidemiological studies on the relationship between climatic conditions and the spread of SARS-CoV-2. Wang et al. [12] studied the relationship between the global daily average temperature and the cumulative number of confirmed COVID-19 cases, and showed that temperature can change the spread of SARS-CoV-2. Therefore, it is recommended that countries and regions with lower temperatures should adopt the most stringent control measures to prevent future outbreaks [12]. Another study encompassing data from 166 countries further found that a temperature increase of 1 °C was associated with a 3.08% decrease in new cases per day and a 1.19% decrease in new deaths per day. A 1% increase in relative humidity is associated with a 0.85% decrease in daily new cases and a 0.51% decrease in daily new deaths [13].

As we all know, the prevalence of diseases is related to many factors. India and Hainan are similar in latitude and climate. There are several factors in the COVID-19 pandemic in India. First, according to the data obtained from the World Air Quality Index project (https://aqicn.org), the air pollution in the COVID-19 pandemic area in India is more serious than in Hainan, and air pollution is conducive to the spread of COVID-19 [14]. Secondly, the population density of India has reached 455 people/km^2^ [15], and the population density of Hainan is 290 people/km^2^ [16]. The population density of India is much higher than that of Hainan. High population density is a catalyst for the spread of COVID-19 and its subsequent spread [17]. According to the safety guidelines of the World Health Organization, a person should keep a distance of more than 1 m from people who cough and sneeze, but this becomes very difficult in densely populated areas. India’s highly dense slum areas are more susceptible to infection, because people living there are in close contact with each other under unsanitary conditions and cannot obtain sufficient safe water and sanitation facilities [18]. Limited space, low sanitation standards, and shared community facilities (faucets, public toilets, etc.) make the people in slum areas vulnerable to the infection and spread of COVID-19. Finally, India’s medical conditions are worse than China’s [19]. The continuous increase of COVID-19 patients naturally makes India’s treatment of COVID-19 patients not very timely, thereby exacerbating the spread of COVID-19.

In relation to this case study the average temperature in Wuhan from January to February 2020 was between 4.4 °C and 9 °C, and the average humidity was between 86% and 79%. In the same period, the average temperature of Haikou that the patient arrived in was 20.7°C, and the average humidity there was 82% and 83% in January and February, respectively; while in Dongfang City, Hainan Province, the average temperature was 22.2°C and the average humidity there was 77% and 76% in January and February, respectively. It can be seen intuitively from the meteorological data that compared with the two cities in the tropics, Wuhan has similar humidity but extremely different temperatures. The above reminds once again that the higher temperature in the tropics has an impact on the spread and infectious ability of SARS-CoV-2, which is consistent with the results of previous studies on climate and the infectiousness of the new coronavirus [6–10].

One poignant factor seen with this patient was the rapid progression after the initial onset of the disease, which quickly resulted in severe illness, and eventual death. This is consistent with study of Wu et al. [20] which showed that after studying the imported and local COVID-19 that occurred in Hainan, that the clinical manifestations and outcomes of imported cases that occurred in Hainan, a tropical area, were much more serious than local cases. The above findings have certain value for the clinical treatment of COVID-19 cases that occur in the tropics.

In the autopsy of this patient, we found that the damage of SARS-CoV-2 to the human body was mainly concentrated in the lungs, and manifested as diffuse damage to the lung tissue mainly due to the destruction of alveolar structure, but the lung manifestation in this case have an onset stage. The repeated virus tests performed in this patient’s autopsy 14 d before death and after treatment were all negative. According to the “COVID-19 diagnosis and treatment plan” issued by the National Health Commission of the People’s Republic of China at that time, the patient’s COVID-19 had been cured. However, the virus test was positive only in the unconventionally extracted lower respiratory tract and respiratory lavage fluid 4 d and 2 d before death. The swab test results of the secretions and body fluids of multiple organs and parts extracted during the dissection showed that no virus was detected in other parts except the lungs. The presence of coronavirus in the lung tissue was confirmed by an electron microscope image showing the characteristic virus form. From an aetiological point of view, it was confirmed that the patient was a clinical case of an initial negative test result becoming positive. It also illustrates that the residue of SARS-CoV-2 in the deep tissues of the lungs may be the possible cause of the change from negative nucleic acid results to positive in clinical patients. These findings are consistent with the research results of Yao et al. [21]. Patients with COVID-19 who are about to be discharged from the hospital should be tested for SARS-CoV-2 in the alveolar lavage fluid in as many cases as possible to ensure that the lung lesions have been completely cured, so as to avoid recurrence.

As Yao et al. [21] indicated, although we have a lot of research on COVID-19, research on pathological changes need to be increased, especially since cases in which SARS-CoV-2 turn from negative to positive are rare. The deceased patient reported in this study showed mild symptoms, but died of sudden cardiac arrest. The characteristics of lung lesions are also different from the case in our report. The differences are COVID-19 general lesions, secondary to severe purulent infection, and the formation of abscesses. However, in the lung tissues, we did not find the common hyaline membrane formation seen in other reports of COVID-19 infection, which may be related to secondary infection. Further staining was carried out on the lung tissue to confirm that the pulmonary interstitium and the interstitial vascular adventitia were hyperplasia of collagen fibres. A large amount of exuded matter from the alveolar cavity was mainly protein mucus, and some were neutral or acidic proteins. The special staining examination also showed focal fungal hyphae and spore structure, indicating that fungal pathogens were involved in the secondary infection. During the examination, we found that the patient had haemorrhage in the left pleural cavity, and a large adherent blood clot was seen in the lower lobe of the left lung, which was consistent with haemorrhage during her lifetime. In addition, no fractures of ribs, sternum, thoracic vertebrae, etc., were found. There were no puncture holes or other injuries in the bilateral chest wall, and no clinical operation records such as thoracentesis in the clinical medical records. It is speculated that in the COVID-19 disease state, due to the extensive and severe lung lesions, the texture becomes hard and brittle, and it loses the elasticity and toughness seen under normal conditions. The lungs are caused by chest compressions during the dying rescue process. The traction caused a small rupture of the severely diseased lung tissue. This is the first reported case of pleural haemorrhage secondary to COVID-19 infection, which is of reference significance for clinical treatment.

The significant change found in this case is that lymphocytes in lymphatic organs and tissues including spleen, tonsils, lymph nodes, and gastrointestinal tissues were sparse and extremely reduced, indicating that the patient’s systemic lymphatic system was already in a state of extreme failure. Combined with the immunostaining of the hilar lymph nodes, the lymph follicles in the hilar lymph nodes were atrophied and the number decreased. The number of T lymphocytes, helper T lymphocytes, and cytotoxic T lymphocytes stained by CD3, CD4, and CD8 decreased significantly. The number of CD20 stained B lymphocytes was significantly reduced. CD138 staining showed that individual plasma cells were scattered in the alveolar wall and lung interstitium. CD68 staining showed that in addition to scattered macrophages in the alveolar wall and lung interstitium, scattered focal macrophages were also seen in the alveolar cavity. CD56 staining indicated that the number of natural killer cells was significantly reduced. The above observations illustrate from another aspect that the severely ill COVID-19 patient had a severe collapse of the immune system function in the late stages.

In this case, we performed SARS-CoV-2 virus nucleic acid detection on the glutaraldehyde electron microscope preservation solution and formalin-fixed tissues for 14 d, and no viral nucleic acid was detected. This indicated that the specimen tissues of COVID-19 patients are no longer infectious after being preserved by the above methods, and can be submitted for inspection and preservation as routine tissues.

In summary, the autopsy findings of this case of COVID-19 and the analysis of the medical history will help to improve our understanding of a disease that poses an unprecedented threat to human health. More autopsy examination cases are needed to confirm whether the characteristics displayed are common.

## Authors’ contributions

Jie Cai and Bo Wang analysed data and drafted manuscript. Tao Song, Peng Zhang and Ren Long worked as the anatomy participants. Xiaoran Liu gathered the data. Jianqiang Deng and Jianhua Chen conducted the study and critical revision of the manuscript for important intellectual content. All authors contributed to the final text and approved it.
